# Coenzyme Engineering of a Hyperthermophilic 6-Phosphogluconate Dehydrogenase from NADP^+^ to NAD^+^ with Its Application to Biobatteries

**DOI:** 10.1038/srep36311

**Published:** 2016-11-02

**Authors:** Hui Chen, Zhiguang Zhu, Rui Huang, Yi-Heng Percival Zhang

**Affiliations:** 1 Biological Systems Engineering Department, Virginia Tech, 304 Seitz Hall, Blacksburg, Virginia 24061, USA; 2 Cell Free Bioinnovations Inc. 1800 Kraft Drive, Suite 222, Blacksburg, VA 24060, USA; 3 Tianjin Institute of Industrial Biotechnology, Chinese Academy of Sciences, 32 West 7^th^ Avenue, Tianjin Airport Economic Area, Tianjin 300308, China

## Abstract

Engineering the coenzyme specificity of redox enzymes plays an important role in metabolic engineering, synthetic biology, and biocatalysis, but it has rarely been applied to bioelectrochemistry. Here we develop a rational design strategy to change the coenzyme specificity of 6-phosphogluconate dehydrogenase (6PGDH) from a hyperthermophilic bacterium *Thermotoga maritima* from its natural coenzyme NADP^+^ to NAD^+^. Through amino acid-sequence alignment of NADP^+^- and NAD^+^-preferred 6PGDH enzymes and computer-aided substrate-coenzyme docking, the key amino acid residues responsible for binding the phosphate group of NADP^+^ were identified. Four mutants were obtained via site-directed mutagenesis. The best mutant N32E/R33I/T34I exhibited a ~6.4 × 10^4^-fold reversal of the coenzyme selectivity from NADP^+^ to NAD^+^. The maximum power density and current density of the biobattery catalyzed by the mutant were 0.135 mW cm^−2^ and 0.255 mA cm^−2^, ~25% higher than those obtained from the wide-type 6PGDH-based biobattery at the room temperature. By using this 6PGDH mutant, the optimal temperature of running the biobattery was as high as 65 °C, leading to a high power density of 1.75 mW cm^−2^. This study demonstrates coenzyme engineering of a hyperthermophilic 6PGDH and its application to high-temperature biobatteries.

Biobatteries are a type of enzyme-catalyzed fuel cells that can directly convert chemical energy of a fuel into electricity in a closed system^1^,^2^,^3^. In biobatteries, electrons transfer from chemical compounds to electrodes via two means: mediated electron transfer (MET) via coenzymes (e.g., NAD^+^) and electron mediators (e.g., benzyl viologen, vitamin K_3_), which often produce high current and power densities; and direct electron transfer (DET) featuring high over-potential, mediator-free, and simple configuration^4^. Although sugar-powered biobatteries have advantages of running on renewable sugary fuels, high safety, high energy density potentials, quiet and mild operating conditions, three main technical challenges include low power density, short lifetime, and high cost, hindering its potential applications^5^,^6^.

The discovery and utilization of thermostable enzymes from thermophilic microorganisms are of importance to increase enzyme stability and potentially decrease enzyme purification cost. If used in biobatteries, these thermophilic enzymes can often lead to prolonged lifetime of enzymes^7^,^8^,^9^. *Thermotoga maritima* is an anaerobic, rod-shaped eubacterium, originally isolated from geothermally heated marine sediment at Valcano, Italy. Growing in the optimal temperature of ~80 °C, *T. maritima* is regarded as an invaluable source of intrinsically thermostable enzymes^10^. 6-phosphogluconate dehydrogenase (6PGDH, E.C.1.1.44), the third enzyme in the pentose phosphate pathway, converts 6-phophogluconate and NADP^+^ to ribulose 5-phosphate, NADPH and CO_2_. The thermostable 6PGDH from *T. maritima* is very stable with a half-life time of 140 h at 80 °C, and can be easily purified by heat precipitation^10^. However, this enzyme prefers NADP^+^ to NAD^+^ so that it has not been tested in biobatteries that should work on NAD^+^ as the coenzyme.

Rational design is one of the protein engineering approaches to engineer enzymes based on knowledge of their structures and catalytic mechanisms^11^,^12^. It may offer a rapid solution to tailor enzymes with desired properties. In bioelectrocatalysis, enzyme kinetics, stability, and electron transfer efficiency can be enhanced by rational design methods, including mutating several key amino acids, trimming off nonessential protein fragments, shuffling domains, and modifying protein surface^13^,^14^. Coenzyme engineering that changes coenzyme selectivity (i.e., NAD^+^ or NADP^+^) of oxidoreductases is one of the important tools in metabolic engineering, synthetic biology, and biocatalysis. Changing the coenzyme selectivity of dehydrogenases from NADP^+^ to NAD^+^ is highly desirable because (1) NAD^+^ is less costly than NADP^+ ^15,16 and (2) NADH is more stable than NADPH^17^,^18^,^19^. For biobatteries, changing coenzyme preference to NAD^+^ must be important because NADP^+^ cannot be efficiently used to generate electricity^8^,^20^. Intensive studies have been conducted to change or broaden coenzyme selectivity of redox enzymes from NADP^+^ to NAD^+ ^21,22,23,24. However, there are few studies pertaining to the coenzyme engineering of thermostable dehydrogenases with the application to biobatteries^25^.

In this study, we demonstrated to switch coenzyme preference of *T. maritima* 6PDGH (*Tm*6PGDH) from NADP^+^ to NAD^+^ by rational design. The best *Tm*6PGDH mutant was applied in the biobattery, exhibiting an improved performance and a high power density at the elevated temperature.

## Results

### Amino acid-sequence alignment and structure analysis of 6PGDH

**Figure 1:**
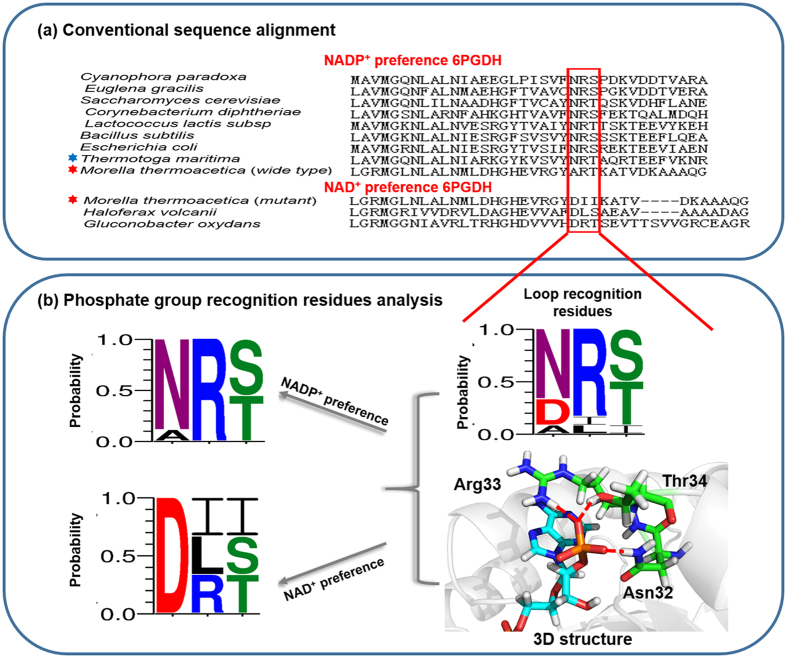
(**a**) Amino acid sequence alignment of the coenzyme-binding motif of various 6PGDH enzymes. The residues composing the loop region and responsible for coenzyme recognition are boxed. Red stars represent *M. thermoacetica* wide-type NADP^+^-preferred 6PGDH and NAD^+^-preferred 6PGDH mutant. Blue star indicates *T. maritima* 6PGDH studied in this research. (**b**) Subalignments of key amino acid residues playing an important role in 2′–phosphate interaction. Colors in sequence logo refer to hydrophobic (black), positive charge (blue), negative charge (red) and polar (green) residues.

We analyzed the Rossmann fold domain of two kinds of 6PGDHs based on their coenzyme specificity (Fig. 1a). Most 6PGDHs in nature favor NADP^+^, such as those from *Syanophora paradoxa* (B2NIW2), *Euglena gracilis* (B2NIV9), *Saccharomyces cerevisiae* (P38720), *Corynebacterium diphtheria* (Q6NHC5), *Lactococcus lactis* (P96789), *Bacillus subtilis* (P80859), *E. coli* K12 (P00350), *T. maritima* (WP_004081528.1) and *M. thermoacetica* (WP_025774778.1). On the other hand, NAD^+^-preferred 6PGDHs are rare, including those from *Haloferax volcanii* (D4GST8), *Gluconobacter oxydans* (G5EBD7) and engineered *M. thermoacetica* 6PGDH. The alignment of the loop region shows that three amino acids (positions 32, 33, and 34) in NADP^+^-preferred 6PGDHs are highly conservative (Fig. 1b), which are asparagine, arginine and threonine (Asn32, Arg33 and Thr34) in *Tm*6PGDH. Via NADP^+^ docking, these residues are found in close contact with the 2′-phosphate of the adenosine ribose via hydrogen bonds (Fig. 1b). Additionally, Arg33 is able to form a cation-phosphate interaction with the NADP^+^ adenosine ring, further stabilizing the NADP-enzyme complex. However, NAD^+^-preferred 6PGDH enzymes have the acidic aspartate residue in the N-terminal end of loop region, which is also very conservative and completely different with that of NADP^+^ preferred-6PGDH. The other two residues exhibit three different combinations. According to the above information, we hypothesized that the changes in Asn32, Arg33, and Thr34 could enable *Tm*6PGDH to work on NAD^+^. Consequently, three different mutants (i.e., N32D, N32D/R33I/T34I and N32D/R33L/T34S) were firstly designed as mutation candidates.

### Structural basis of coenzyme preference

**Figure 2:**
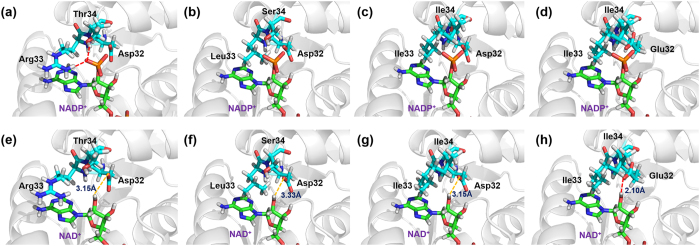
The representations of enzyme-coenzyme interactions. (**a**) Interaction analysis of mutant N32D with NADP^+^; (**b**) Interaction analysis of mutant N32D/R33L/T34S with NADP^+^; (**c**) Interaction analysis of mutant N32D/R33I/T34I with NADP^+^; (**d**) Interaction analysis of mutant N32E/R33I/T34I with NADP^+^; (**e**) Interaction analysis of mutant N32D with NAD^+^; (**f**) Interaction analysis of mutant N32D/R33L/T34S with NAD^+^; (**g**) Interaction analysis of mutant N32D/R33I/T34I with NAD^+^; (**h**) Interaction analysis of mutant N32E/R33I/T34I with NAD^+^. The residues involved in defining coenzyme-specificity are shown as sticks. Hydrogen bonding between residues and cofactor were shown as red line. The distance between the two atoms were shown as yellow line. The figures were made using PyMol.

To identify key amino acids pertaining to coenzyme preference, a detailed analysis of the coenzyme-6PGDH complexes was performed based on the auto-docking program. The mutant N32D has the replacement of the alkaline asparagine with the acidic aspartate, deleting the former hydrogen bond between the 2′-phosphate of NADP^+^ and the asparagine residue of the wide type *Tm*6PGDH (Fig. 2a) and therefore resulting in a decrease in the binding affinity of the mutant N32D towards NADP^+^. To further enhance coenzyme preference of N32D, the amino acids with large and hydrophobic side chain (leucine and isoleucine) were introduced to the position 33 and 34 to construct the mutant N32D/R33L/T34S, N32D/R33I/T34I. The newly-introduced residues can further disrupt the other two original hydrogen bonds with 2′-phosphate of NADP^+^. Moreover, their large and hydrophobic side chains display much stronger steric exclusion effect and compress the space used to accommodate the 2′-phosphate group of adenosine monophosphate moiety of NADP^+^ (Fig. 2b,c). The combined effect of the deletion of original hydrogen bond and the enhanced steric exclusion should lead to a significant decrease in binding with NADP^+^ for the enzyme.

To better bind NAD^+^, it is also important to form productive hydrogen bonds. In our case, the distance between the hydrogen atom in 2′-hydroxyl group of NAD^+^ and the oxygen atom in carboxyl group of Asp32 in the mutant N32D and N32D/R33I/T34I is calculated as 3.15 Å (Fig. 2e,g), which equals to the threshold value of the hydrogen bond formation^26^. Therefore, their ability to bind NAD^+^ could be improved slightly. In the mutant N32D/R33L/T34S (Fig. 2f), however, such distance increases to 3.33 Å, which is too long to form a productive hydrogen bond. Consequently, this mutant may have no positive effect on NAD^+^ binding. Instead, when the residue aspartate at position 32 is mutated to a similar one, glutamate, this distance can be decreased to 2.10 Å (Fig. 2h). In this new mutant N32E/R33I/T34I, a new hydrogen bond is formed, increasing the binding with NAD^+^.

### Enzyme kinetics analysis

According to the alignment result and structure analysis, the plasmids encoding mutants N32D, N32D/R33L/T34S, N32D/R33I/T34I and N32E/R33I/T34I were constructed by site-directed mutagenesis. The protein mutants were overexpressed in *E. coli* BL21 (DE3) and purified to homogeneity based on SDS-PAGE (data not shown). The kinetic constants on NADP^+^ and NAD^+^ of the wild type and mutant 6PGDHs are summarized in Table 1. The mutant N32D has a 460-time higher *K*_*m*_ value of 2.3 mM on NADP^+^ and a slightly decreased *K*_*m*_ value of 4.0 mM on NAD^+^ compared with those of the wide type. The triple-site mutant N32D/R33I/T34I exhibits a far higher *K*_*m*_ value of 69.5 mM on NADP^+^ and a slightly decreased *K*_*m*_ value of 3.9 mM on NAD^+^. Another mutant N32D/R33L/T34S has a *K*_*m*_ value on NADP^+^ of more than 100 mM. Its *K*_*m*_ value on NAD^+^ is also increased to 7.5 mM. However, these three mutants do not show significant differences of *k*_*cat*_ on NADP^+^ with that of the wild-type.

**Figure 3:**
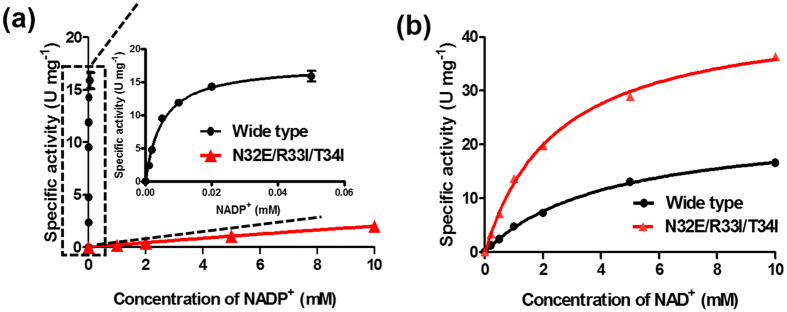
The kinetic parameters towards (**a**) NADP^+^ and (**b**) NAD^+^ of the wide-type or mutant 6PGDH (N32D/R33I/T34I).

The mutant N32E/R33I/T34I was found to be the best one. As shown in Table 1 and Fig. 3, an almost 2-fold declined *K*_*m*_ (from 4.3 mM to 2.5 mM) and a 2-fold increased *k*_*cat*_ on NAD^+^ (from 23.3 to 47.9 s^−1^) compared to that of wide type can be observed. The catalytic efficiency (*k*_*cat*_/*K*_*m*_) towards NADP^+^ decreases from 3,520 to 0.2 s^−1^, while the catalytic efficiency towards NAD^+^ increases from 5.4 to 19.2 s^−1^. Overall, the coenzyme catalytic coefficient ratio of NAD^+^ to NADP^+^ increases about 6.4 × 10^4^ times for this mutant.

### Biobattery tests

**Figure 4:**
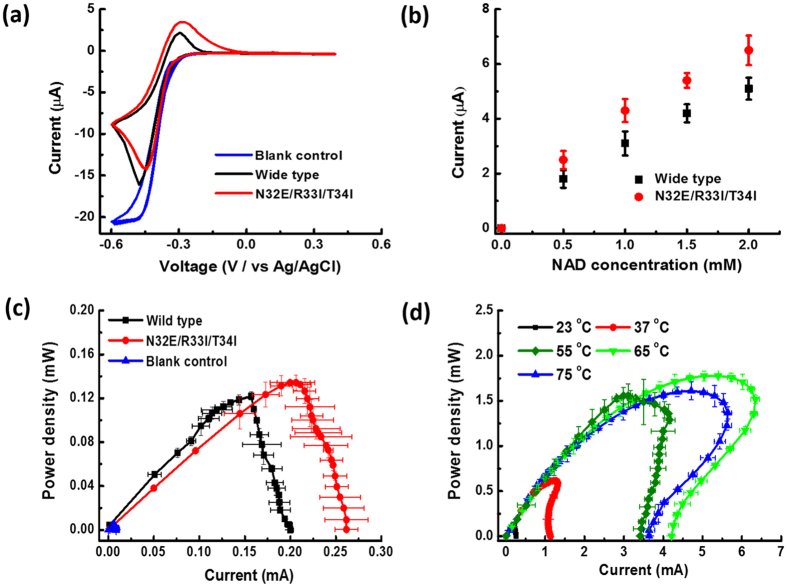
Electrochemical performances of the wild-type 6PGDH and mutant (N32D/R33I/T34I)-based biobatteries. (**a**) Cyclic voltammetry; (**b**) Peak current from cyclic voltammetry with the mutant or wild-type 6PGDH versus the concentration of NAD^+^; (**c**) Power curves of the biobattery at 23 °C; (D) Power of the biobattery equipped with the mutant 6PGDH at a temperature from 23 to 75 °C.

The effect of the best mutant (N32E/R33I/T34I) versus the wild type 6PGDH was evaluated electrochemically in an anodic reaction system containing two enzymes: 6PGDH and DI, a coenzyme (NADP^+^ or NAD^+^), an electron mediator AQDS, and a 6-phosphogluconate substrate (6PG). Cyclic voltammetry results clearly show that both 6PGDHs produce significant oxidation current peaks at −0.3 V versus Ag/AgCl. The mutant N32E/R33I/T34I exhibits a current density 25% higher than that generated by the wild type (Fig. 4a). The blank control samples including the one without the fuel, without the NAD^+^, or with denatured 6PGDH did not generate any oxidation current peak. The current densities of 6PG oxidation at various NAD^+^ concentrations were further evaluated, by plotting peak currents at −0.3 V versus Ag/AgCl against NAD^+^ concentrations varying from 0 to 2 mM (Fig. 4b). The current density increases along with the increase of the NAD^+^ concentration, while the mutant generates currents 20–30% higher than those by the wild-type. Both figures suggest that the mutant N32E/R33I/T34I is more effective than the wild-type on using NAD^+^ as the coenzyme in the electrochemical reaction system.

Moreover, both wide-type enzyme and mutant N32E/R33I/T34I were compared in biobatteries in terms of power output using linear sweep voltammetry. The whole-cell biobattery consisted of a carbon nanotube-coated carbon felt anode and a commercial membrane electrode assemble stacked in pile. An increased loading of 6PG, NAD^+^, AQDS, and 6PGDH was applied in order to get a high power output of the biobattery. The two polarization curves represent that the maximum power density and current density of the biobattery catalyzed by the mutant 6PGDH are 0.135 mW cm^−2^ and 0.255 mA cm^−2^ respectively at the room temperature, while for the biobattery catalyzed by the wild type 6PGDH, they are 0.122 mW cm^−2^ and 0.200 mA cm^−2^ (Fig. 4c). At elevated temperatures, the mutant 6PGDH-powered biobattery significantly increased power outputs of 0.61 mW cm^−2^ at 37 °C, 1.58 mW cm^−2^ at 50 °C, 1.75 mW cm^−2^ at 65 °C, and 1.58 mW cm^−2^ at 75 °C (Fig. 4d). It is noted that the biobattery has an optimal power density at 65 °C, probably due to the optimal enzyme kinetics and the increased mass transfer at this temperature. Too high temperature caused the deactivation of diaphorase, resulting in decreased power outputs in the biobattery.

## Discussion

Most NAD(P)^+^-dependent dehydrogenases have the Rossmann fold, whose loop region contains three conservative amino acids. Alkaline amino acids, such as asparagine and arginine, in the first two positions play a critical role in NADP^+^-preferred 6PGDHs through the formation of stable hydrogen bonds with 2′-phosphate of NADP^+ 27–29^. In contrast, NAD^+^-preferred 6PGDH enzymes have acidic aspartate residues in the N-terminal end of loop region (Fig. 1b). The substrate docking result (Fig. 2) indicates that the formation of a new hydrogen bond with NAD^+^ through the replacement of alkaline amino acid by acidic one is favorable to increase the enzyme activity on NAD^+^, and even reverse the coenzyme preference from NADP^+^ to NAD^+^. Moreover, our previous research on the directed evolution of *Moorella thermoacetica* 6PGDH also demonstrated that the introduction of an acidic amino acid, aspartate, in the loop of the Rossmann fold to form a new hydrogen bond with 2′-hydroxyl group of NAD^+^ resulted in a 4,300-fold reversal of coenzyme selectivity from NADP^+^ to NAD^+ ^30. Reports on many other dehydrogenases also confirmed the positive effect of the formation of new hydrogen bond in reversing the coenzyme preference^21^,^24^,^30^,^31^,^32^,^33^,^34^.

Our results of kinetic constants of the wild-type enzyme and the mutant N32E/R33I/T43I are in good agreement with the structure analysis. For the mutant N32E/R33I/T43I, the coenzyme binding ability of 6PGDH on NADP^+^ is decreased greatly, because the mutant loses its original three hydrogen bonds with the 2′-phosphate of NADP^+^ and suffers from a strong steric exclusion effect from the hydrophobic side chain of two newly introduced isoleucines (Fig. 2d). Those significant structural changes result in a 15,800-fold increase in *K*_*m*_ on NADP^+^ and a 17,600-fold decline in catalytic efficiency (*k*_*cat*_/*K*_*m*_) on NADP^+^ (Table 1). While on NAD^+^, the introduced acidic residue, glutamate, improves the binding of coenzyme due to the formation of the new productive hydrogen bond between Glu32 and 2′-hydroxyl group of NAD^+^. A 2-fold decline in *K*_*m*_ and a 4-fold increase in catalytic efficiency on NAD^+^ compared to wide type *Tm*6PGDH are observed (Table 1). As a result, the overall coenzyme catalytic coefficient ratio of NAD^+^ to NADP^+^ of this mutant increases about 6.4 × 10^4^ times.

As a thermostable enzyme with a half-life time of 140 h at 80 °C, 6PGDH from *T. maritima* has great potential in biobattaries. Firstly, it has a prolonged lifetime of biobattaries due to high stability of the enzymes^35^. Secondly, it allows to run biobattaries at elevated temperatures or tolerate harsh conditions, such as in deserts or extremely hot conditions. Third, high-temperature biobattaries would further generate high power density because of increased enzyme activity^8^,^10^ and mass transfer^36^. To our limited knowledge, this present biobattery is the one running on the second highest temperature among all the EFCs reported. The highest temperature of EFC was 80 °C by using a thermostable alcohol dehydrogenase but its current density was only 0.21 mA cm^−2^ (5% of this study)^37^.

In conclusion, this work reports a coenzyme specificity change of *Tm*6PGDH through rational design with its application to biobatteries. Via enzyme sequence alignment and structure analysis of the coenzyme-enzyme complexes, the key amino acid residues that are responsible for binding coenzymes were identified. The coenzyme preference was reversed from NADP^+^ to NAD^+^ through site-directed mutagenesis. The optimal temperature of the biobattary with the mutant was as high as 65 °C, resulting in a high power density of 1.75 mW cm^−2^.

## Methods and Methods

### Chemicals, Bacterial Strains and Medium

All chemicals were reagent grade or higher, purchased from Sigma-Aldrich (St. Louis, MO) or Fisher Scientific (Pittsburgh, PA), unless otherwise noted. *E. coli* Top10 was used for general molecular cloning and *E. coli* BL21 (DE3) was used for recombinant protein expression. All enzymes for molecular biology experiments were purchased from New England Biolabs (NEB, Ipswich, MA). Strains, plasmids, and oligonucleotides primers used in this study are listed in Table 2.

Cetyltrimethylammonium bromide (CTAB) and glutaraldehyde (GA) were purchased from Sigma-Aldrich (Saint Louis, MO). 9,10-anthraquinone-2,7-disulphonic acid (AQDS) was purchased from Pfaltz & Bauer (Waterbury, CT). Carbon felt (C100 AvCarb) was purchased from Fuel Cell Earth (Woburn, MA). COOH-functionalized multi-wall carbon nanotubes (CNTs) with an outer diameter of <8 nm and a length of 10–30 μm were purchased from CheapTubes.com (Brattleboro, VA). Membrane electrode assemblies (MEAs) consisting of the Nafion 212 membrane and a carbon cloth cathode modified with 0.5 mg cm^−2 ^Pt were purchased from Fuel Cell Store (San Diego, CA). The glassy carbon (GC) working electrode (3 mm diameter), Ag/AgCl reference electrode, Pt wire counter electrode were bought from BASi (West Lafayette, IN).

### Site directed mutagenesis

The *Tm*6PGDH gene was amplified from plasmid^10^ by a primer pair of 6PGDH_IF and 6PGDH_IR. pET20b vector backbone was amplified with a primer pair of 6PGDH_VF and 6PGDH_VR. Plasmid pET20b-*6pgdh* based on the two DNA fragments was obtained using a Simple Cloning method^38^.

The QuickChange^TM^ site-directed mutagenesis method (Stratagene, La Jolla, CA) was used to introduce point mutations into the *Tm*6PGDH sequence according to the protocol of the NEB Phusion site-directed mutagenesis kit. The mutant N32D, N32D/R33I/T34I, N32D/R33L/T34S, and N32E/R33I/T34I were prepared by their respective primer pairs (Table 2). PCR reaction solution (50 μL) containing 1 ng of plasmid template (pET20b-*6pgdh*) was conducted as follows: 98 °C denaturation for 1 min; 20 cycles of 98 °C denaturation for 30 s, 60 °C annealing for 30 s and 72 °C extension for 2.5 min; and 72 °C extension for 5 min. The PCR product was digested by DpnI followed by the purification of gel electrophoresis using a Zymoclean™ Gel DNA Recovery Kit (Zymo Research, Irvine, CA) and then transformed into *E. coli* BL21 (DE3).

### Overexpression and purification of wide-type *Tm*6PGDH and mutants

The transformants were grown overnight at 37 °C in Luria-Bertani medium plates containing 100 μg mL^−1^ ampicillin. For liquid culture, once A_600_ of the cultured cell reached ~0.8, 0.2 mM IPTG was added to induce protein expression at 37 °C for 6 h. Cell pellets were harvested by centrifugation and then were re-suspended in a 20 mM sodium phosphate and 0.3 M NaCl buffer (pH 7.5) containing 10 mM imidazole. After sonication and centrifugation, the His-tagged protein in the supernatant was loaded onto the column packed with HisPur Ni-NTA Resin (Fisher Scientific, Pittsburgh, PA) and eluted with 20 mM sodium phosphate buffer (pH 7.5) containing 300 mM NaCl and 250 mM imidazole. Mass concentration of protein was determined by the Bradford assay using bovine serum albumin (BSA) as the standard.

### *Tm*6PGDH activity assay

The activities of wide type *Tm*6PGDH and mutants were measured in 100 mM HEPES buffer (pH 7.5) with the final concentration of 2 mM 6-phosphogluconate (6PG), 2 mM NAD(P)^+^, 5 mM MgCl_2_ and 0.5 mM MnCl_2_ at 70 °C for 5 min. The formation of NAD(P)H was measured at 340 nm by a UV/visible spectrophotometer (Beckman Coulter, Fullerton, CA). The enzyme unit was defined as one μmole of NAD(P)H produced per min. To determine enzyme kinetic parameters on coenzymes, the enzyme activity was measured in the same buffer above except changing the concentration of NAD(P)^+^ (2 μM to 10 mM). The result was regressed by GraphPad Prism 5 (Graphpad Software Inc., La Jolla, CA) and the apparent *K*_*m*_ and *k*_*cat*_ for NAD(P)^+^ of wide type and mutant *Tm*6PGDH were given based on Michaelis-Menten nonlinear regression. All the reactions contained three independent triplicates and the data were fitted within the linear range.

### Electrochemical measurement

All electrochemical measurements were conducted using a 600D Potentiostat from CH Instruments Inc. (Austin, TX) interfaced to a PC. The reaction solution was flushed by argon gas for 20 minutes before each measurement. Each measurement was conducted in triplicate.

Cyclic voltammetry was performed in the anodic compartment at a scan rate of 20 mV s^−1^ using a 3-electrode system with a GC working electrode, an Ag/AgCl reference electrode and a Pt wire counter electrode. The anolyte contained up to 2 mM NAD^+^, 5 mM 6PG, 2.5 mM AQDS, 100 mM HEPES (pH 7.3), 10 mM MgCl_2_, 0.5 mM MnCl_2_, 100 mM NaCl, 0.0133 mg mL^−1^ of mutated or wild type *Tm*6PGDH, and 0.015 mg mL^−1^ of *Geobacillus stearothermophilus* diaphorase (DI).

Linear sweep voltammetry was performed at a scan rate of 5 mV s^−1^ in a stacked fuel cell configuration. A 1-cm^2^ carbon felt (CF) was coated with 0.25 mL of CNTs solution (CNTs: CTAB: H_2_O = 1 mg: 1 mL: 1 mL) and 30 μL of GA, dried at the room temperature, and used as the anode. An air-breathing carbon cathode was coated with 0.5 mg cm^−2 ^Pt. A Nafion 212 membrane was used to separate two electrodes. The anolyte contained increased loadings of 8 mM NAD^+^, 40 mM 6PG, 16.7 mM AQDS, 0.05 mg mL^−1^ of mutated or wild type *Tm*6PGDH, and 0.06 mg mL^−1^ of DI. The performance of the biobattery with mutated *Tm*6PGDH was further evaluated at increased temperatures up to 75 °C.

### Sequence alignment

Manually annotated and reviewed sequence data for 6PGDH were retrieved from the National Center for Biotechnology Information and Uniprot Database^39^. Clustal Omega^40^,^41^ was used to perform a multiple sequence alignment. The sequence logo plot^42^ was created with the WebLogo 3.3 interface^43^.

### Structure analysis

The three-dimensional (3D) structure modeling of the wild-type *Tm*6PGDH and mutants were built by SWISS-MODEL based on the *Lactococcus lactis* 6PGDH (PDB: 2IYP) as a template (sequence identity: 46.5%). The final model was validated using the PROCHECK program^44^. The structures of NADP^+^ and NAD^+^ were built by using Chemdraw (PerkinElmer, Waltham, MA). The conformation space of the corresponding coenzyme binding area was analyzed using the Autodock program (Scripps Research Institute, La Jolla, CA).

## Additional Information

**How to cite this article**: Chen, H. *et al*. Coenzyme Engineering of a Hyperthermophilic 6-phosphogluconate Dehydrogenase from NADP^+^ to NAD^+^ with Its Application to Biobatteries. *Sci. Rep.* **6**, 36311; doi: 10.1038/srep36311 (2016).

**Publisher’s note:** Springer Nature remains neutral with regard to jurisdictional claims in published maps and institutional affiliations.

## Figures and Tables

**Table 1:** Kinetics parameters of *Tm*6PGDH and its mutants.

Mutations	*K*_*m*_ (mM)		*k*_*cat*_ (s^−1^)		*k*_*cat*_/*K*_*m*_ (mM^−1^*s^−1^)		Ratio *k*_*cat*_/*K*_*m*_
	NADP^+^	NAD^+^	NADP^+^	NAD^+^	NADP^+^	NAD^+^	NAD^+^/NADP^+^
Wide-type	0.005 ± 0.0004	4.3 ± 0.4	17.6 ± 0.7	23.3 ± 2.0	3520	5.4	1.5 × 10^−3^
N32D	2.3 ± 0.1	4.0 ± 0.1	16.7 ± 1.0	29.3 ± 1.2	7.3	7.3	1
N32D/R33L/T34S	>100	7.5 ± 0.8	ND	25.4 ± 1.4	ND	3.4	ND
N32D/R33I/T34I	70 ± 17	3.9 ± 0.4	15.9 ± 5.8	32.2 ± 2.2	0.2	8.3	41.4
N32E/R33I/T34I	79 ± 20	2.5 ± 0.2	15.9 ± 7.9	47.9 ± 2.2	0.2	19.2	96

Each value represents the average of three independent measurements. ND: Undetectable.

**Table 2:** The strains, plasmids, and oligonucleotides used in this study.

Description	Contents	Reference/sources
**Strain**		
*E. coli* Bl21^star^(DE3)	B F^–^ *ompT gal dcm lon hsdS*_*B*_(*r*_*B*_^–^*m*_*B*_^–^) *rne131* (DE3)	Invitrogen
*E. coli* TOP10	F– *mcr*A cr*mrr*-*hsd*RMS-*mcr*BC) Φ80*lac*Zac80 Δ*lac*X74 *rec*A1 *ara*D139 Δ(*ara leu*) 7697 *gal*U *gal*K *rps*L (StrR) *end*A1 *nup*G	Invitrogen
**Plasmid**		
pET20b		Invitrogen
pET20b-*6pgdh*	Amp^R^, *6pgdh* expression cassette containing *Tm*6PGDH protein	in this study
primers*		
6PGDH_IF	5′-TTAACTTTAAGAAGGAGATATACATATGAAATCCCACATTGGCCTGATCG-3′	*Thermotoga maritima*
6PGDH_IR	5′-AGTGGTGGTGGTGGTGGTGCTCGAGGCCAATCTCCCCCTCCTCCCAGTTG-3′	
6PGDH_VF	5′-CAACTGGGAGGAGGGGGAGATTGGCCTCGAGCACCACCACCACCACCACT-3′	pET20a
6PGDH_VR	5′-CGATCAGGCCAATGTGGGATTTCATATGTATATCTCCTTCTTAAAGTTAA-3′	
N32D_F	5′-AAAGTGAGCGTGTAT**GAC**CGTACTGCCCAGCGT-3′	pET20a-*6pgdh*
N32D_R	5′-ACGCTGGGCAGTACG**GTC**ATACACGCTCACTTT-3′	
N32D/R33I/T34I_F	5′-GTGAGCGTGTATGAC**ATTATT**GCCCAGCGTACAGAA-3′	pET20a-*6pgdh* N32D
N32D/R33I/T34I_R	5′-TTCTGTACGCTGGGC**AATAAT**GTCATACACGCTCAC-3′	
N32D/R33L/T34S_F	5′-GTGAGCGTGTATGAC**CTGAGC**GCCCAGCGTACAGAA-3′	pET20a-*6pgdh* N32D
N32D/R33L/T34S_F	5′-TTCTGTACGCTGGGC**GCTCAG**GTCATACACGCTCAC-3′	
N32E/R33I/T34I_F	5′-GTGAGCGTGTAT**GAG**ATTATTGCCCAGCGTACAGAA-3′	pET20a-*6pgdh* N32D/R33I/T34I
N32E/R33I/T34I_R	5′-TTCTGTACGCTGGGCAATAAT**CTC**ATACACGCTCAC-3′	

*Boldface nucleotide sequences indicate mutation positions.
